# How low can you go? Tracking eye movements during reading at different sampling rates

**DOI:** 10.3758/s13428-025-02713-3

**Published:** 2025-06-09

**Authors:** Bernhard Angele, Zeynep Gunes Ozkan, Marina Serrano-Carot, Jon Andoni Duñabeitia

**Affiliations:** 1 Centro de Investigación Nebrija en Cognición (CINC), Universidad Nebrija, Calle de Asura, 90, 28043 Madrid, Spain; 2 ERI-Lectura and Department of Methodology, Faculty of Psychology, Universitat de Valéncia, Av. de Blasco Ibáñez, 21, 46010 Valencia, Spain

**Keywords:** Reading, Eye movements, Sampling rate, Word count

## Abstract

Eye-movement research has revolutionized our understanding of reading, but the use of eye-tracking techniques in investigating the reading process is still limited by the cost of high-precision eye-tracking, which limits research to laboratories with sufficient resources. It is important to evaluate to what extent cognitive processes during reading can be measured with less expensive eye-tracking devices. One such way may be to use devices with a lower sampling rate, which are much less expensive than high-sampling rate eye-trackers. We recorded readers’ eye movements during reading at different sampling rates and show that it is possible to measure the classic effect of word frequency on fixation duration, reflecting ongoing cognitive processing during reading, at sampling rates ranging from 250 to 2000 Hz. We simulate even lower sampling rates and show that, with a sufficiently large sample size, it is possible to detect the effect of word frequency even at very low sampling rates (30–125 Hz). Our results demonstrate that, in principle, low sampling rates are not an obstacle to studying the effects of cognitive processing during reading.

It has now been approximately 40 years since infrared-based eye trackers revolutionized the study of cognitive processes during reading (for a summary, see e.g., Rayner, 1998, 2009). In this time, eye-tracking technology has evolved from tracking the Purkinje reflections on the outside and inside of the eyes using photodiodes (Cornsweet & Crane, 1973; Crane & Steele, 1985; Evans & Gutmann, 1978; Young & Sheena, 1975) to video-based methods used by modern eye-trackers such as the EyeLink system by SR Research (Hutton, 2019). Most recently, there have also been very promising attempts to collect eye-movement data online (Kaduk et al., 2023; Papoutsaki et al., 2016). Despite the technological progress, eye trackers can still be characterized by two fundamental properties: their spatial accuracy and their temporal accuracy. Carter and Luke (2020) further distinguish between spatial accuracy and precision, where accuracy refers to the difference between the measured gaze position and the true gaze position (the systematic error) and precision refers to whether the eye tracker provides consistent measurements, i.e., the random error in the system (see Reingold, 2014, for an example of a technique that can measure precision). Both measures are usually given in degrees of visual angle.

For example, a recent study by Ehinger et al. (2019) found that the EyeLink 1000 system had an average spatial accuracy (i.e., difference between the gaze target participants were asked to fixate and the recorded fixation location) of 0.57° of visual angle and a precision of 0.023°^1^, while a glasses-based competitor system from Pupil Labs (now marketed as Pupil Labs Core) had a slightly higher error of 0.82° of visual angle and a lower precision of 0.119°. Note that Ehinger et al. (2019) used the EyeLink 1000 without head stabilization. The accuracy and precision may be better in setups where the head is supported by a chinrest.

The precision of an eye-tracking system is determined by many factors such as the optical properties of the camera and the resolution of the image sensor, while the accuracy depends on the calibration of the system. During the calibration process, participants are typically asked to fixate a series of targets on the screen, with the resulting measurements being used to establish a correspondence between eye and head orientation and gaze location, and to detect any inconsistencies in this association over the different calibration targets. A good calibration can improve accuracy significantly, giving experimenters a degree of control over quality of their data.

For temporal accuracy, this relationship is quite different. Here, the technical limitations of the system, not the calibration, are what mostly determines the quality of the data that can be collected. Just as every image sensor records data at a set spatial resolution, it is limited in terms of how many images it can record per second. This property is known as *sampling rate* and differs greatly between eye-tracking systems. The SR Research EyeLink 1000 system, for example, can record at up to 2000 Hz (i.e., one sample every 0.5 ms), while the older EyeLink II is limited to 500 Hz (i.e., one sample every 2 ms). All of these systems have been used in a large number of studies on eye movements during reading. It is important to note that sampling rate is a hard technical limitation that is difficult to overcome and that high-speed image sensors that also feature a high spatial resolution are very expensive. Indeed, most eye-tracking systems are quite expensive, and more affordable systems typically have vastly lower maximum sampling rates than more expensive systems.

This causes sampling rate to act as a bottleneck for many eye-tracking applications. For example, Carter and Luke (2020) state that systems with lower sampling rates are more appropriate for studies focused on where participants look, implying that the question of when participants move their eyes needs to be investigated using systems with higher sampling rates. Most studies of eye-tracking during reading use eye trackers with sampling rates upwards of 500 Hz (the majority of studies in the last ten years have used sampling rates of at least 1000 Hz). However, this (mostly unwritten) requirement means that many opportunities to investigate eye movements during reading are lost. For example, researchers in developing countries often lack the funding to buy an eye tracker that can record at 1000 or even just 500 Hz, but might be able to purchase a more affordable system. In the last decades, most eye-tracking studies on reading have been conducted in a small number of countries – mostly in Western Europe and North America, but, recently, also in China – with a limit number of languages and populations being studied (Angele & Duñabeitia, 2024). We suspect that the cost of high-speed and precision eye-tracking systems has been a major contributor to this situation. The high cost of eye-tracking systems has also prevented their wide adoption outside of research laboratories, limiting their potential uses in studying reading in natural settings. In particular, the use of eye trackers in schools is usually limited to a single device that is temporarily set up for a particular research study and then taken back to the laboratory (this was done, for example, by Duñabeitia et al., 2013). The routine use of eye-trackers in classrooms, particularly the monitoring of learning progress in developing readers, could provide crucial insights into how children learn to read and highlight teachers to the need of interventions. However, schools having their own eye-trackers and using them continually to monitor reading development in children is virtually unheard of because of the significant cost involved. Because of this, we lack the knowledge about how individual reading development trajectories are reflected in eye movements and which eye-movement patterns might be diagnostic of reading difficulties that may be addressed by timely interventions.

Schools are not the only area where a wider application of eye-tracking technologies for the study of reading might be beneficial. Eye-tracking is widely used to evaluate advertising, but this is mostly limited to rough estimates of where a viewer allocates their attention rather than analyzing the reading process in detail and using word-based measures. In such applications, the use of affordable eye-tracking devices with low sampling rates is very common, but such studies do not analyze the whole eye-movement record. Being able to analyze individual fixations rather than interpreting just a simplified overview such as a heat map could lead to surprising insights even for individual readers. For example, with sufficiently accessible eye-tracking technology, such as an eye tracker built into glasses or a tablet/e-Reader device, individuals could monitor their attentional engagement with the text and avoid periods where attention disengages from the text while the eyes keep moving (for a recent attempt to do this using online eye-tracking data, see Hutt et al., 2023).

In summary, there would be many benefits to using eye-tracking during reading in more contexts and with more populations. However, how can we overcome the sampling rate bottleneck? One possibility would be to wait for affordable devices with high sampling rates to become available. However, given that there currently is not much consumer demand for cameras (e.g., webcams) with frame rates of 1000 Hz or more, this may take many years. The alternative is to investigate, systematically, what phenomena related to cognitive processing during reading can be studied the sampling rates that are currently available. At the lower and mid-price range of the market, dedicated eye-tracking devices usually have sampling rates between 60 Hz (e.g., Tobii Pro Spark or Gazepoint GP3) and 250 Hz (e.g., Tobii Pro Fusion). On the other hand, webcams usually have frame rates of either 30 and 60 Hz, with very few devices featuring 120 Hz. There have been some recent attempts to use webcams for eye-tracking in order to study language processing (Hutt et al., 2023; Slim & Hartsuiker, 2022; Van der Cruyssen et al., 2023; e.g., Vos et al., 2022), but, so far, there are virtually no studies of (sentence) reading using webcam eye-tracking, most likely due to researchers doubting whether the quality of the resulting data is sufficient to obtain meaningful results.

Indeed, what types of effects in reading can be studied with such low-sampling rate devices is an open question. The first step to addressing this question is to determine what sampling rate is required to accurately detect saccades. Most measures in reading research are based on either saccades or fixations (which are defined as the pauses between saccades) and therefore rely on accurate saccade detection. On a fundamental level, the Nyquist–Shannon sampling theorem (Shannon, 1949) states that a signal can be accurately reconstructed from its samples if the sampling rate is at least twice the highest frequency present in the signal. The question is what the relevant frequency of the saccade signal is. Bahill et al. (1981) analyzed the velocity power spectrum of saccades and found that its bandwidth (i.e., the range of frequencies in the saccade velocity signal that contains a significant proportion of its information) is around 74 Hz, i.e., virtually all the information necessary to accurately estimate saccade velocity is contained in the frequencies up to 74 Hz (Bahill et al., 1982). This means that, according to the Nyquist-Shannon sampling theorem, a sampling frequency of 148 Hz should be enough to detect saccades with a velocity-based algorithm, although Bahill et al. (1982) include a safety margin and recommend a sampling rate of 333 Hz, suggesting that the most commonly used sampling rate of 1000 Hz is most certainly more than what is necessary to accurately detect saccades. Of course, recording at sampling rates lower than this limit of 148 Hz does not necessarily mean that saccades detection is impossible; rather, lower sampling rates will introduce measurement error as the gaze position cannot be updated until the next sample is taken. In reading research, we are mostly interested in estimating fixation duration, which requires the estimation of both the end time of the saccade preceding the fixation and the start time of the next saccade. Andersson et al. (2010) showed that the sampling errors for these two time points often cancel each other out at least partially. They found that if we calculate the mean of multiple fixation time estimates, this error reduces further as a function of the number of data points. This means that sample size (number of participants and trials) can potentially compensate for low sampling rate in terms of sampling error.

Of course, even the presence of sampling error in the fixation duration estimates does not mean that we cannot obtain useful data. In the present study, we will take a practical approach to the question of whether reading can be studied at low sampling rates: We will attempt to determine which is the lowest sampling rate that allows us to find evidence of cognitive processing on eye movements during reading. For this, we need a benchmark effect – a phenomenon that is well-studied and whose existence (and effect size) is clear.

In eye movements during reading, the word frequency effect on fixation duration is ideal for this. Word frequency is one of the most important variables not just in the study of reading, but in all of cognitive and experimental psychology (Brysbaert et al., 2011, 2017). The frequency of a word in a language corpus, traditionally measured in occurrences per million, can be interpreted as a stand-in for the number of times a participant has encountered a word, i.e., as a learning effect (Brysbaert et al., 2017). Word frequency is correlated with many other variables such as word length, age of acquisition, word predictability, etc. Nevertheless, there are consistent effects of word frequency even when these (and many other) variables are statistically controlled (e.g., Brysbaert & Cortese, 2011). Importantly, the word frequency effect is not linear. There is a “saturation effect” such that differences in processing speed and accuracy between low-frequency words are much more pronounced than differences between high-frequency words. For example, processing of a word with a frequency of 1/million is substantially slower than processing of a word with a frequency of 2/million, while words with a frequency of 100/million and 101/million will be nearly indistinguishable in terms of processing difficulty. Because of this, Brysbaert et al. (2017) recommend the use of the logarithmic Zipf scale^2^ to model word frequency effects in reading experiments. In reading, the effect of word-frequency effect on eye movements was first noted by Erdmann and Dodge in 1898 (Erdmann & Dodge, 1898; see also Huey, 1908). They found that readers make more pauses (fixations) for difficult material than for easy and familiar material.

Today, the word frequency effect is usually measured using fixation duration (Rayner, 1998). From the gaze position data recorded by the eye-tracking device, we detect fixations and match the fixation position to the position of words on the screen. We can then calculate specific aggregated fixation time measures such as first fixation duration (FFD, the duration of the first fixation on each word), gaze duration (GD, the duration of the first fixation plus any subsequent refixations on a word), and total viewing time (TVT, the sum of all fixations on a word). The word-frequency effect can be measured experimentally. In this case, we construct sentence frames that are compatible with both a low and a high-frequency target word. For example, the sentence frame The slow waltz/music captured her attention (Rayner & Duffy, 1986) can be presented either with the low-frequency target word waltz or with the high-frequency target word music. The word frequency effect is then calculated as the difference between the average value of an aggregated fixation time measure (FFD, GD, TVT, and others) across the low-frequency target words (e.g., waltz) and the average value of the same measure on the high-frequency target words (e.g., music).

The size of this word frequency effect depends on the characteristics of the target words chosen and of the population studied. For example, Rayner and Duffy (1986) found a frequency effect of 37 ms in FFD and 87 ms in GD, while Inhoff and Rayner (1986) estimated the effect as 16 ms in FFD and 29 ms in GD. In addition to experiments with an experimental word frequency manipulation, there have been eye-tracking corpus studies collecting a large amount of data and attempting to predict fixation time measures on all words in a sentence based on the properties of each word, sentence, and, potentially, participant. The first such study was done by Schilling et al. (1998), followed by the Potsdam Sentence Corpus (Kliegl et al., 2004, 2006) and many other similar projects, most notably the Provo Corpus (Luke & Christianson, 2017), the bilingual Ghent Eye-Tracking Corpus (Cop et al., 2015, 2016) and, most recently, the Multilingual Eye-Movement Corpus (MECO, Siegelman et al., 2022).

Overall, the word frequency effect in reading is extremely well established and has been replicated numerous times across different languages and tasks. There is no doubt that it exists, and we have a reasonably precise estimate of its effect size. It is therefore the ideal benchmark effect to investigate how recording eye movements at different sampling rates affects our ability to detect evidence of cognitive processing in the eye-movement record. In this study, we recorded the same participants reading sentences with a target word frequency manipulation during the same experimental session at four different sampling rates (250, 500, 1000, and 2000 Hz) and calculated the most commonly used fixation time measures of FFD, GD, and TVT. Additionally, we simulated even lower recording sampling rates that were unavailable on our eye tracker (31.25, 60, and 125 Hz), both by removing samples (“drop” algorithm) and by averaging over samples (“average” algorithm). If recording sampling rate does not matter in terms of detecting the effects of a fundamental variable such as word frequency in cognitive processing on eye movements during reading, we would expect to find a word frequency effect in all of these measures and in all of the recording sampling rates, both on the target word as a consequence of the word frequency manipulation and on all of the words in the sentence in a corpus-style analysis. We would also not expect to see differences in the size of these frequency effects. If the sampling rate does impact our ability to detect the effects of cognitive processing on the eye-movement record, we might find that lower recording sampling rates are associated with more noise and thereby reduce the statistical power we have to detect the word frequency effect. In this case, we might find evidence of the word frequency effect at higher sampling rates, but not at lower ones, and may be able to provide estimates of the sample size needed to detect an effect at different sampling rates.

## Method

We report here how we determined our sample size, all data exclusions (if any), all manipulations, and all measures in the experiment.

### Participants

Thirty-five undergraduate students from Nebrija University, aged from 18 to 35 years (mean age 22.3; 26 identifying as female and nine identifying as male), participated in this study in exchange for a small compensation (24€ for approximately two hours of participation). Three participants were unable to complete the experiments due to problems with eye tracker calibration. All participants were native Spanish speakers, reported normal or corrected-to-normal vision and no previous diagnosis of reading disorders and were naïve as to the purpose of the study. All the participants gave informed consent before the experiment. This research followed the principles and guidelines of the Declaration of Helsinki, and we obtained ethical approval from the Nebrija University Research Ethics Committee (Ref. UNNE-2023-0031).

#### Rationale for sample size

Based on the rule of thumb recommended for small effect sizes (15 ms) by Brysbaert and Stevens (2018), we aimed to collect at least 1600 data points per condition, which, given that we recorded 100 sentences at each of the four sampling rates and that 50 of these sentences would be displayed in each of two-word frequency conditions, translates to 32 participants in total.

**Table 1:** Sentence properties by target word frequency condition. Note that, to show the distributions of the properties, this table gives the median and the interquartile range (range between the 25th and the 75th percentile)

		Target word frequency condition	
Characteristic	Overall *N* = 800^*1*^	high *N* = 400^*1*^	low *N* = 400^*1*^
Words per sentence	11 (9, 13)	11 (10, 13)	11 (9, 13)
M(zipf) (all words)	5.70 (5.45, 5.86)	5.73 (5.50, 5.91)	5.66 (5.42, 5.81)
M(number of letters, all words)	5.36 (4.80, 5.91)	5.36 (4.80, 5.92)	5.33 (4.80, 5.89)
Target zipf frequency	3.87 (3.24, 3.99)	3.99 (3.93, 4.81)	3.24 (3.12, 3.38)
Target number of letters	8.00 (7.00, 10.00)	8.00 (7.00, 10.00)	8.00 (7.00, 10.00)
Target position (characters)	29 (21, 39)	29 (21, 39)	29 (21, 39)
Acceptability rating	4.47 (4.09, 4.69)	4.53 (4.19, 4.70)	4.40 (4.00, 4.69)

^*1*^Overall column includes both low- and high-frequency conditions (*N* = 800). Numbers in parenthesis are first and third quantiles

This table does not include sentences that were excluded from the analysis

### Materials

We selected 440 pairs of low- and high-frequency nouns from the EsPal corpus (Duchon et al., 2013). We used the LexOPS package (Taylor et al., 2020) in the R statistical software (R Core Team, 2024) to match the nouns based on frequency while controlling for length (range between 3 to 16), gender, and number. Based on Brysbaert et al. (2017)’s findings that the word frequency effect for the average reader is strongest in the range between 3 and 5 Zipf (1 and 100/million, see above), we selected 220 words with higher frequencies (mean Zipf = 4.30, SD = 0.51) for the high-frequency condition, and 220 lower frequency words (mean Zipf = 3.28, SD = 0.24) for the low-frequency condition.

In the following step, sentences were generated for the noun pairs, ensuring that the target word occurred near the middle of each sentence and that the sentence context preceding the target word was identical for low- and high-frequency target words. The context following the target words was allowed to vary in order to maintain sentence coherence. For example, for the word pair vestida (high frequency, “dressed”) and trémula (low frequency, “trembling”), the sentence for vestida was Al mirarla, noté que estaba vestida elegantemente y con muchoestilo. (“When I looked at her, I noticed that she was dressed elegantly and with a lot of style”). The sentence for trémula was Al mirarla, noté que estaba trémula e inquieta (“When I looked at her, I noticed that she was trembling and restless”).

We used GPT-3.5-Turbo (OpenAI, Inc.) through the *openai* package for R (Rudnytskyi, 2023) to generate initial versions of the sentences, which were then carefully inspected by Spanish native speakers who identified unacceptable sentences and corrected them where possible. We selected 400 sentences to include as experimental items. For the acceptability rating task, we additionally selected 40 filler sentences that were judged to be not acceptable by native speakers.

Table 1 shows the number of words, mean Zipf frequency, mean number of letters, Zipf frequency of the target word, number of letters of the target word, position of the target word in the sentence, and mean acceptability rating for each sentence by frequency condition.

### Apparatus

An SR Research EyeLink Portable Duo video-based eye tracker (SR Research Ltd., Canada) was used to record participants’ eye movements while reading sentences with four different sampling rates. Sentences were presented on a 24-inch BENQ XL2430 LCD monitor with a refresh rate of 144 Hz using a computer running the OpenSesame software (Version 4.0.13, Mathôt et al., 2012) with the PyGaze plugin (Dalmaijer et al., 2013) on Ubuntu Linux 22.04. Viewing and recording were binocular, but, in line with the majority of eye-tracking studies during reading, we analyzed only data from the right eye. During the experiment, participants were seated approximately 60 cm from the monitor with their head on a chin rest to reduce head movements.

### Procedure

The experiment took place in a quiet room. Participants were told they would be presented with individual sentences and asked to read them silently to evaluate their acceptability. They were instructed to read each sentence carefully and ensure that they understood them and could make an acceptability judgment before ending the trial. At the beginning of the experiment, participants completed ten practice trials to familiarize themselves with the procedure. The experiment consisted of four blocks of 110 trials each (including ten unacceptable sentences as targets for the acceptability rating task), with a block duration of about 20–25 min. Participants were encouraged to take short breaks between blocks to avoid fatigue. During each block, eye movements were recorded at one of four different sampling rates (250, 500, 1000, and 2000 Hz). Sampling rate condition and block order were counterbalanced, as was the frequency condition of the items within the blocks, ensuring that each participant saw each item exactly once and that each item was seen equally often in each sampling rate and frequency condition and block in the experiment. Within each block, items were presented in random order.

At the beginning of each block, the eye-tracker was calibrated using the built-in EyeLink nine-point calibration. This procedure was repeated whenever needed. Each trial started with a drift check at the center of the screen followed by a rectangular gaze target at the left center of the screen. Once participants had fixated the gaze target for 250 ms, the sentence appeared, with the first word positioned where the gaze target had been. Sentences were presented in black 20-point Courier New (monospaced) font on a white background, with each character being 12 pixels wide and subtending about $0.34^{\circ }$ of visual angle. Participants ended trials by directing their gaze to the lower right corner of the screen, where a small fixation target was presented. Following each trial, participants rated sentence acceptability on a scale of “1” (completely unacceptable) to “5” (completely acceptable) using a Black Box Toolkit five-button USB response pad (The Black Box Toolkit, Ltd.). To be able to evaluate participants’ performance on the acceptability rating task, 40 out of the 440 sentences in the experiment contained semantic or grammatical anomalies (see Materials section).

### Data analysis and dependent variables

For our first analysis, we treated the data collected at each of the four sampling rates as separate studies. For each data set, we detected fixations from the gaze samples (see below).

#### EyeLink fixation detection algorithm

In this first set of analyses, fixations were identified based on the output of the saccade-detection algorithm built into the EyeLink system. The EyeLink automatically detects saccades and fixations and saves this information in the EyeLink data files (EDFs) along with the raw x and y gaze samples. The EyeLink algorithm detects saccades based on velocity and acceleration, enabling the user to specify three thresholds to distinguish saccades from fixations: motion (degrees), velocity (degree/s) and acceleration (degree/$\textrm{s}^{2}$). While these thresholds can be adjusted, in practice most researchers use the default settings (Configuration 0: Cognitive) recommended by SR Research for reading and cognitive research. The thresholds applied in this configuration are (1) a velocity threshold of 30 degrees/s, (2) a minimum acceleration threshold of 8000 degrees/$\textrm{s}^{2}$, and (3) a spatial motion threshold of 0.1 degree. The algorithm also has a maximum velocity, which is 60 degrees/s (but tasks involving reading on a monitor do not normally involve such velocities).

#### Data analysis

For each trial, we calculated FFD , GD, and TVT (the sum of all fixations on a word) for all the words in the sentence, including the target word. In the first analysis, we fitted Bayesian linear mixed models, using the *brms* package (Bürkner, 2017, 2018, 2021) in R^3^, for each sampling rate and each dependent measure. We limited these analyses to the target word, which was the word with the frequency manipulation. We log-transformed the fixation time measures to reduce the effect of outliers on the distribution and used the Gaussian family in *brms* to model them. All models included the frequency condition as a categorical fixed effect, with “high frequency” coded as -0.5 and “low frequency” coded as 0.5 (Schad et al., 2020). We included the maximum random effects structure (i.e., random slopes for the frequency effect) for each model (Barr et al., 2013). We used weakly informative priors (a Gaussian distribution with a mean of 0 and an SD of 10) for the regression coefficients, and the default priors set by *brms* for all other parameters. Each model was fitted using four chains with 5000 iterations each, for which 1000 were warm-up iterations. The models converged successfully (all $\hat{R}$s = 1.00). We report the mean, the estimates (*b*) and the 95% Bayesian credible intervals (95% CI^4^) based on the posterior distribution of each parameter. To simplify the interpretation of the posterior distribution, we will assume that there is evidence for an effect if 0 is not a credible value for its coefficient (i.e., if it is not part of the 95% CI).

For the second set of analyses, we combined all the data from the four blocks for each dependent measure and fit a model with frequency condition and sampling rate as categorical fixed effects as well as their interaction. As sampling rate had four levels, we used three orthogonal contrasts to make comparisons between these models: Contrast 1 compared the lower sampling rates (250 and 500 Hz) on one side with the higher sampling rates (1000 and 2000 Hz) on the other side, Contrast 2 compared 250 Hz with 500 Hz, and Contrast 3 compared 1000 Hz with 2000 Hz^5^. These models also had the maximum random effects structure (i.e., random slopes for frequency, sampling rate, and the interaction over subjects and sentences).

##### Downsampling and fixation detection

The EyeLink Portable Duo only offers sampling rates of 250, 500, 1000, and 2000 Hz. In order to explore the detectability of frequency effects at even lower sampling rates, we performed a second set of analyses in which we reduced the sampling rate of the raw data (with samples consisting of gaze position, *x*-position, and *y*-position) artificially using a downsampling algorithm. We compared two algorithms: (1) the “drop” algorithm, which removed most samples from the data and only kept every *n*-th sample in order to simulate a lower sampling rate, and the “average” algorithm which averages over every *n* samples. For example, in order to downsample 500 Hz data to 50 Hz, the “drop” algorithm would remove nine out of every ten samples in the data and only keep every 10th sample, while the “average” algorithm would replace every group of ten samples in the data with one new sample that is the average (in terms of time, *x*-position, and *y*-position) of the ten samples. Each of these algorithms represents a different assumption about how low-sampling rate devices work compared to high-sampling rate devices. The “drop” algorithm assumes that low-sampling rate devices obtain high-accuracy samples, just at a lower rate. The “average” algorithm assumes that lower sampling-rate devices do not obtain one precise estimate of gaze position, but rather “blur” together all the gaze positions during one sampling cycle. This may be a more realistic assumption given how digital cameras work. As the EyeLink fixation detection algorithm is built into the EyeLink Host PC software and cannot be run on arbitrary data, this downsampling process made it impossible for us to use the EyeLink algorithm for saccade detection. Because of this, we applied the saccade detection algorithm proposed by Engbert and Kliegl (2003) to the downsampled raw data. The Engbert & Kliegl (EK) algorithm is a velocity-based algorithm, as it was implemented in the *saccades* package (von der Malsburg, 2019) in the R statistical software (R Core Team, 2024). This algorithm detects saccades-based velocity, with the velocity threshold set to 30 degrees/s (the same as the default setting of the EyeLink algorithm). We used the default settings of the algorithm as implemented in the *saccades* package. We used these algorithms to downsample all trials to 125, 50, and 31.25 Hz, detected saccades, and calculated fixation time measures as described above. We then fitted the same Bayesian linear mixed models as described above to the downsampled data.

## Results

### Data quality

In Table 2 we report data quality measures (mean validation error as a measure of accuracy, the root mean square of sample-to-sample differences as a measure of precision, and the percentage of samples that were lost due to blinks and overall) for each sampling rate according to the guidelines proposed by Dunn et al. (2023). In line with the findings by Blignaut and Beelders (2012), the root mean square of the sample-to-sample distances was lower for higher sampling rates. This is because, at lower sampling rates, there is more time for movement between samples. The other measures did not vary between sampling rates and also did not differ between experimental conditions.

**Table 2:** Data quality measures for each sampling rate

	Accuracy	Precision	Data loss	
Sampling rate	Error	S2S RMS	Total	Blinks
(Hz)	(deg)	(deg)	(%)	(%)
250	0.48	0.50	4.43	3.33
500	0.50	0.25	4.32	2.87
1000	0.44	0.15	4.40	3.16
2000	0.47	0.11	4.32	2.98

For accuracy, error denotes the average difference between calibration and validation in degrees of visual angle across all calibrations. S2S RMS is the root mean square of the sample-to-sample distances in degrees of visual angle across all trials. Total data loss is the total percentage of samples lost (including during blinks) across all trials. Blink data loss is the percentage of samples lost during events that were labeled as blinks by the EyeLink algorithm across all trials

### Acceptability

As expected, participants rated most of the experimental sentences as highly acceptable (mean = 4.34, SD = 0.49), while the filler sentences were rated much lower (mean = 3.57, SD = 0.89). Table 1 shows acceptability ratings for the 400 sentences included in the analysis.

### Global measures

Table 3 shows the number of subjects and trials recorded for each sampling rate. It also shows the number of fixations detected by the EyeLink algorithm, both overall and per trial, as well as the number of fixations merged and excluded because they were under 80 ms (fixations that were under 80 ms and within 12 pixels of a longer fixation were merged into the longer fixation; all other fixations under 80 ms were excluded), over 800 ms, or occurred within 100 ms of a blink. As these criteria sometimes overlap, the table also shows the total number of fixations excluded and the total percentage of fixations excluded.

**Table 3:** Number of subjects and experimental trials, total number of fixations (fix, detected by the EyeLink algorithm), fixations per trial, and number of fixations excluded due to short fixation times (and, of these, number of short fixations that were merged into another fixation instead of being excluded), long fixations times, and proximity to blinks for each sampling rate as well as the total number and percentage of fixations excluded

	Sampling rate in Hz			
	250	500	1000	2000
Number of subjects	32	32	32	32
Number of trials	3,200	3,200	3,200	3,200
Number of fix	46,897	46,662	46,445	45,687
Mean fix per trial	14.66	14.6	14.54	14.31
Fix < 80 ms	1454	1362	1431	3000
Fix < 80 ms (merged)	29	37	70	440
Fix > 800 ms	69	111	126	45
Fix before or after blink	3256	2892	2516	2413
Total fix excluded	4283	3941	3735	5068
Percent of fix excluded	9.13	8.45	8.04	11.09

This table does not include the non-acceptable trials

Overall, both the number of fixations detected and the number of fixations excluded did not differ substantially between sampling rates. However, there were more short fixations detected (and excluded) in the 2000-Hz data set than for the other three sampling rates. On the other hand, there were fewer long fixations detected for the 2000-Hz sampling rate. The reason for this may be that the saccade detection algorithm may not be optimized for a 2000-Hz sampling rate and may sometimes split longer fixations into shorter ones. Another interesting observation is that more fixations were detected to be close to blinks in the 250-Hz data set than for the other sampling rate. This may simply be due to the lower temporal resolution.

**Table 4:** Mean and standard error for first fixation duration (FFD), gaze duration (GD), and total viewing time (TVT) on the target word by frequency condition and sampling rate

	*FFD*		*GD*		*TVT*	
	Mean	SE	Mean	SE	Mean	SE
*250 Hz*						
High frequency	242	2.12	330	4.07	477	7.77
Low frequency	245	2.12	369	4.98	551	8.63
Effect	3		39		73	
*500 Hz*						
High frequency	238	2.10	326	4.27	473	8.01
Low frequency	246	2.31	357	5.06	537	8.88
Effect	8		31		64	
*1000 Hz*						
High frequency	234	2.07	317	4.05	467	7.94
Low frequency	244	2.12	362	5.26	532	8.69
Effect	10		44		65	
*2000 Hz*						
High frequency	230	2.00	311	3.86	437	7.09
Low frequency	236	2.13	340	4.60	496	8.17
Effect	7		29		58	

All measures in ms

### Frequency effect

**Fig. 1:**
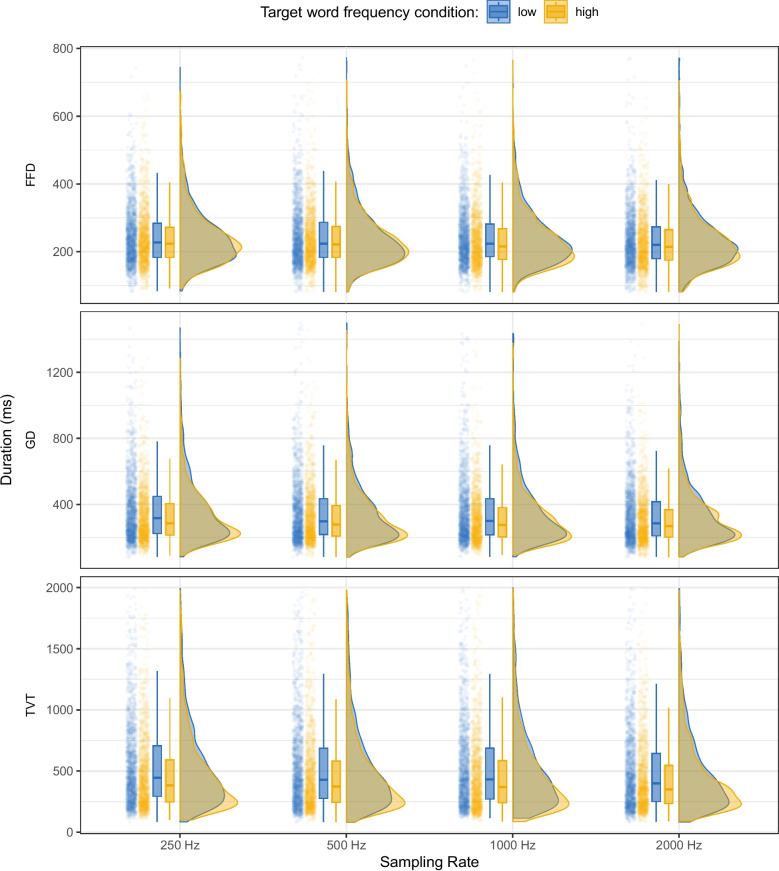
Raincloud plot showing the distributions of first fixation duration (FFD), gaze duration (GD), and total viewing time (TVT) on the target word for low- vs. high-frequency target words across the four sampling rates (250, 500, 1000, and 2000 Hz). For each sampling rate, the left side of the plot shows a jittered point cloud and boxplots (representing median and inter-quartile range, IQR), while the right side shows half-violin plots of the distribution density

For each sampling rate, we calculated first fixation duration (FFD), gaze duration (GD), and total viewing time (TVT) on the target word for the 400 sentences included in the analysis. In addition to the individual fixation exclusions above, we also excluded very high gaze durations (GD > 1500 ms) and total viewing times (TVT > 2000 ms). The maximum number of observations excluded was in TVT at 500 Hz, with 51 of 3026 observations excluded (1.69%). Table 4 shows the mean and standard error for FFD, GD, and TVT on the target word by frequency condition as well as the effect of the frequency manipulation in ms, while Fig. 1 shows the distributions of FFD, GD, and TVT on the target word in the high- and low-frequency conditions across the four sampling rates. As can be seen, the distributions were quite similar across the sampling rates and we found a frequency effect in all sampling rates, however, this effect was very small in FFD.

**Table 5:** Coefficients and 95% credible intervals from Bayesian linear mixed models fitted to log FFD, GD, and TVT calculated from data collected at 250, 500, 1000, and 2000 Hz

	FFD		GD		TVT	
Variable	b	CI	b	CI	b	CI
*250 Hz*						
(Intercept)	**5.45**	**5.40, 5.49**	**5.75**	**5.69, 5.82**	**6.08**	**5.97, 6.19**
Frequency	0.005	-0.020, 0.030	**0.083**	**0.047, 0.119**	**0.153**	**0.109, 0.197**
*500 Hz*						
(Intercept)	**5.44**	**5.39, 5.49**	**5.73**	**5.65, 5.81**	**6.06**	**5.93, 6.19**
Frequency	**0.027**	**0.000, 0.054**	**0.080**	**0.047, 0.113**	**0.147**	**0.105, 0.189**
*1000 Hz*						
(Intercept)	**5.43**	**5.38, 5.48**	**5.72**	**5.64, 5.80**	**6.05**	**5.92, 6.18**
Frequency	**0.036**	**0.013, 0.060**	**0.095**	**0.060, 0.130**	**0.136**	**0.085, 0.185**
*2000 Hz*						
(Intercept)	**5.40**	**5.35, 5.45**	**5.67**	**5.61, 5.74**	**5.96**	**5.85, 6.07**
Frequency	**0.032**	**0.003, 0.060**	**0.077**	**0.040, 0.113**	**0.121**	**0.075, 0.167**

Abbreviation: CI = 95% credible interval

Coefficients for which the 95% credible interval does not include 0 are shown in bold

Table 5 shows the results of the four Bayesian linear mixed models, one for each sampling rate. In FFD on the target word, we observed an effect of the frequency condition, with longer FFDs for low-frequency words compared to high-frequency words at 1000 Hz (*b* = 0.04, SE = 0.01, 95% CI [0.01, 0.06]) and 2000 Hz (*b* = 0.032, SE = 0.015, 95% CI [0.003, 0.06]). There was not sufficient evidence for the frequency effect at 250 Hz (*b* = 0.01, SE = 0.01, 95% CI [-0.02, 0.03]) as the 95% CI included 0. At 500 Hz, (*b* = 0.0272, SE = 0.0137, 95% CI [2e-04, 0.0539]) the 95% CI did not include 0 and was in the same direction as that for 1000 Hz and 2000 Hz, but the lower bound of the interval was very close to 0. Unlike in FFD, in gaze duration (GD) we observed an effect of the frequency manipulation on the target word for all the sampling rates: 250 Hz (*b* = 0.08, SE = 0.02, 95% CI [0.05, 0.12]), 500 Hz (*b* = 0.08, SE = 0.02, 95% CrI [0.05, 0.11]), 1000 Hz (*b* = 0.09, SE = 0.02, 95% CrI [0.06, 0.13]), and 2000 Hz (*b* = 0.08, SE = 0.02, 95% CI [0.04, 0.11]), with longer GDs for low-frequency words compared to high-frequency words. Similarly, in TVT on the target word, we again were able to find evidence of the effect of the frequency manipulation, with longer TVTs for low-frequency words compared to high-frequency words at all sampling frequencies: 250 Hz (*b* = 0.15, SE = 0.02, 95% CI [0.11, 0.2]), 500 Hz (*b* = 0.15, SE = 0.02, 95% CI [0.1, 0.19]), 1000 Hz (*b* = 0.14, SE = 0.03, 95% CI [0.09, 0.19]), and 2000 Hz (*b* = 0.12, SE = 0.02, 95% CI [0.08, 0.17])).

Overall, the frequency effect was quite robust and consistent across the different sampling rates, with the exception of FFD at 250 Hz. It is important to note that, due to the sampling rates available on the EyeLink Portable Duo, we were not able to test any sampling rate under 250 Hz. As described in the Introduction, most affordable eye-tracking methods have much lower sampling rates. Depending on the device, sampling rates of 125, 60, or even 30 Hz are common, with the latter being the typical frame rate of most webcams. It is therefore important to investigate whether we can detect the frequency effect at even lower sampling rates than those available in the EyeLink system.

#### Analysis across all sampling rates

In order to perform a direct test of the effect of sampling rate, we fitted an additional model on all the target word data from each fixation time measure, including all of the sampling rates. Sampling rate was included as an additional fixed effect along with the interaction between sampling rate and the target word frequency condition.

**Table 6:** Coefficients and 95% credible intervals from Bayesian linear mixed models fitted to log FFD, GD, and TVT calculated from all data, with frequency condition, sampling rate, and their interaction as predictors

	FFD		GD		TVT	
Variable	b	CI	b	CI	b	CI
(Intercept)	**5.43**	**5.39, 5.47**	**5.72**	**5.65, 5.78**	**6.03**	**5.92, 6.15**
Frequency (Fq)	**0.025**	**0.011, 0.039**	**0.084**	**0.064, 0.103**	**0.139**	**0.106, 0.173**
*Sampling rate (Hz)*						
$\le$500 vs $\ge$1000	**-0.014**	**-0.025, -0.004**	**-0.023**	**-0.041, -0.005**	**-0.034**	**-0.060, -0.008**
250 vs 500	-0.005	-0.017, 0.007	-0.020	-0.041, 0.001	-0.020	-0.048, 0.008
1000 vs 2000	-0.015	-0.032, 0.002	-0.017	-0.039, 0.005	**-0.031**	**-0.061, -0.002**
*Interactions*						
Fq:$\le$500 vs $\ge$1000	0.009	-0.003, 0.021	0.001	-0.015, 0.018	-0.011	-0.031, 0.009
Fq:250 vs 500	0.011	-0.007, 0.028	-0.002	-0.025, 0.021	-0.004	-0.031, 0.023
Fq:1000 vs 2000	-0.003	-0.022, 0.017	-0.009	-0.034, 0.016	-0.007	-0.034, 0.019

Abbreviation: CI = 95% credible interval

Coefficients for which the 95% credible interval does not include 0 are shown in bold

In the analysis combining all the data (Table 6), we found an overall effect of word frequency across sampling rates in FFD (*b* = 0.02, SE = 0.01, 95% CI [0.01, 0.04]), GD (*b* = 0.08, SE = 0.01, 95% CI [0.06, 0.1]), and TVT (*b* = 0.14, SE = 0.02, 95% CI [0.11, 0.17]). The overall means of the fixation time measures calculated also differed slightly between sampling rates, which may be due to differences in how the saccade and fixation detection algorithm works for data at different sampling rates. At lower sampling rates, it seems saccades are detected to start slightly later and end slightly earlier, leading to longer estimated fixation durations at lower sampling rates. This difference was evident in FFD (*b* = -0.014, SE = 0.005, 95% CI [-0.025, -0.004]), GD (*b* = -0.023, SE = 0.009, 95% CI [-0.041, -0.005]), and TVT (*b* = -0.034, SE = 0.013, 95% CI [-0.06, -0.008]) when comparing the lower sampling rates (250 and 500 Hz) to the higher sampling rates (1000 and 2000 Hz), but not when comparing 250 to 500 Hz and 1000 to 2000 Hz, except in TVT, where there was evidence for longer TVTs at 1000 Hz compared to 2000 Hz (*b* = -0.031, SE = 0.015, 95% CI [-0.061, -0.002]). There was no credible evidence for a difference in the frequency effect depending on the sampling rate, with all 95% CIs for the interactions including 0.

**Table 7:** Number of subjects and experimental trials, total number of fixations (fix, detected by the Engbert & Kliegl algorithm), fixations per trial, and excluded/merged fixations (see Table 3 for details) in the data downsampled to 125, 50, and 31.25 Hz using the average and drop algorithms

	Simulated sampling rate in Hz					
	Average algorithm			Drop algorithm		
	31.25	50	125	31.25	50	125
Number of subjects	32	32	32	32	32	32
Number of trials	3200	3200	3200	3200	3200	3200
Number of fix	40,316	46,014	49,128	42,480	45,913	48,636
Mean fix per trial	12.81	14.41	15.37	13.32	14.37	15.22
Fix < 80 ms	2565	817	752	2132	656	623
Fix < 80 ms (merged)	6	7	52	3	3	32
Fix > 800 ms	202	95	75	141	112	86
Fix before or after blink	79	564	1654	255	800	1,789
Total fix excluded	2837	1433	2403	2503	1523	2421
Percent of fix excluded	7.04	3.11	4.89	5.89	3.32	4.98

Only one block per subject included

**Table 8:** Data downsampled using the drop algorithm, one block per subject: Mean and standard error for first fixation duration (FFD), gaze duration (GD), and total viewing time (TVT) on the target word by frequency condition and simulated sampling rate

	*FFD*		*GD*		*TVT*	
	Mean	SE	Mean	SE	Mean	SE
*31.25 Hz*						
High frequency	214	2.52	268	3.61	388	6.71
Low frequency	221	2.72	303	4.56	468	8.52
Effect	7		36		80	
*50 Hz*						
High frequency	220	2.20	290	3.80	430	7.27
Low frequency	227	2.32	328	4.73	508	8.64
Effect	7		39		78	
*125 Hz*						
High frequency	231	2.03	312	3.98	465	7.69
Low frequency	236	2.08	350	4.92	545	9.06
Effect	5		38		80	

All measures in ms

**Fig. 2:**
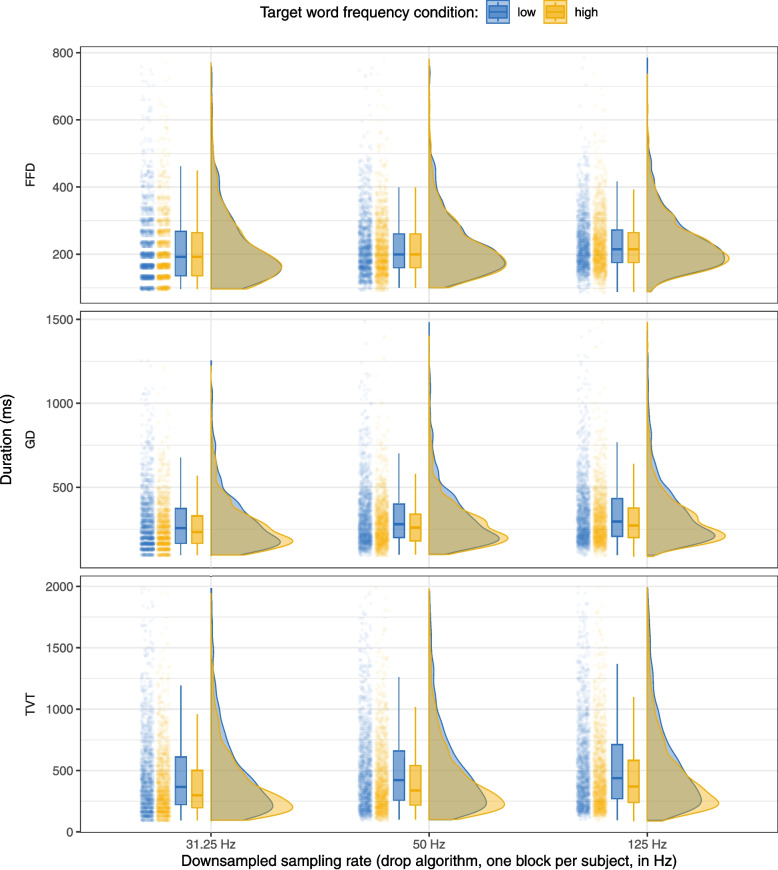
Data downsampled using the drop algorithm, one block per subject: Raincloud plot showing the distributions of first fixation duration (FFD), gaze duration (GD), and total viewing time (TVT) on the target word by frequency condition and simulated sampling rate. For each sampling rate, the left side of the plot shows a jittered point cloud and boxplots (representing median and IQR), while the right side shows half-violin plots of the density distributions

**Table 9:** Coefficients and 95% credible intervals from Bayesian linear mixed models fitted to log FFD, GD, and TVT calculated from data downsampled to 125, 50, and 31.25 Hz using the drop algorithm, with only one block per participant included

	FFD		GD		TVT	
Variable	b	CI	b	CI	b	CI
*31.25 Hz*						
(Intercept)	**5.29**	**5.24, 5.35**	**5.52**	**5.45, 5.60**	**5.85**	**5.72, 5.96**
Frequency	0.031	-0.003, 0.065	**0.100**	**0.054, 0.145**	**0.166**	**0.112, 0.223**
*50 Hz*						
(Intercept)	**5.34**	**5.29, 5.39**	**5.61**	**5.54, 5.68**	**5.96**	**5.84, 6.10**
Frequency	0.029	-0.001, 0.058	**0.099**	**0.056, 0.142**	**0.167**	**0.111, 0.220**
*125 Hz*						
(Intercept)	**5.40**	**5.36, 5.45**	**5.69**	**5.62, 5.76**	**6.04**	**5.92, 6.17**
Frequency	0.016	-0.012, 0.044	**0.087**	**0.046, 0.127**	**0.154**	**0.099, 0.211**

Abbreviation: CI = 95% credible interval

Coefficients for which the 95% credible interval does not include 0 are shown in bold

**Table 10:** Data downsampled using the average algorithm, only one block included: Mean and standard error for first fixation duration (FFD), gaze duration (GD), and total viewing time (TVT) on the target word by frequency condition and simulated sampling rate

	*FFD*		*GD*		*TVT*	
	Mean	SE	Mean	SE	Mean	SE
*31.25 Hz*						
High frequency	211	2.59	263	3.72	377	6.72
Low frequency	221	2.85	304	4.73	462	8.54
Effect	10		41		85	
*50 Hz*						
High frequency	213	2.06	282	3.69	418	7.03
Low frequency	218	2.21	321	4.75	498	8.65
Effect	5		39		80	
*125 Hz*						
High frequency	226	1.96	307	3.87	459	7.52
Low frequency	229	1.97	346	4.88	540	9.00
Effect	4		39		81	

All measures in ms

**Fig. 3:**
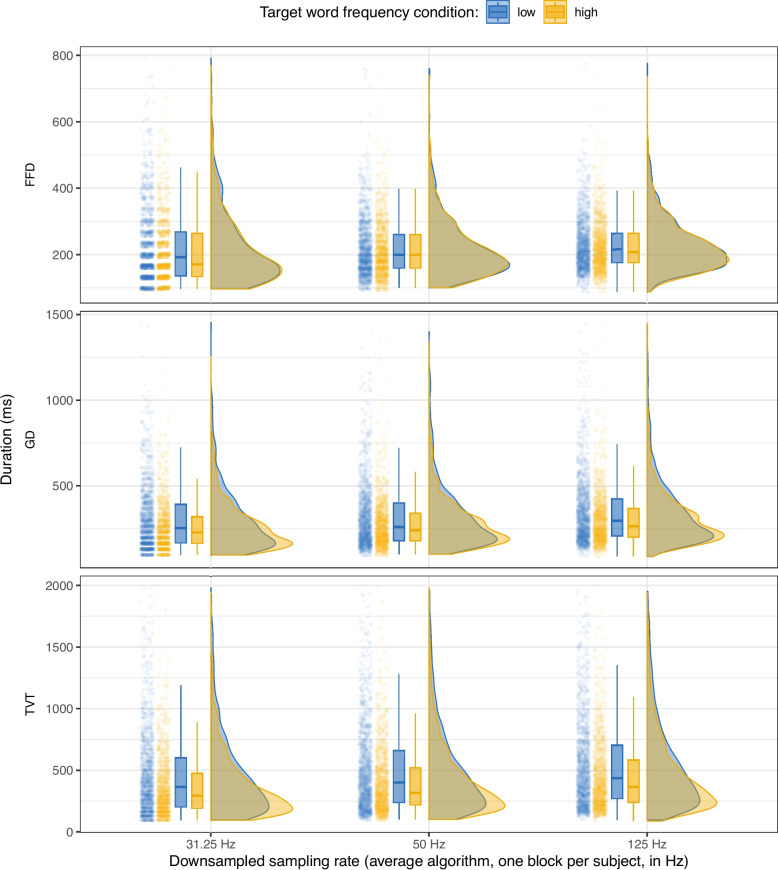
Data downsampled using the average algorithm, one block per subject: Raincloud plot showing the distributions of first fixation duration (FFD), gaze duration (GD), and total viewing time (TVT) on the target word by frequency condition and simulated sampling rate. For each sampling rate, the left side of the plot shows a jittered point cloud and boxplots (representing median and IQR), while the right side shows half-violin plots of the density distributions

**Table 11:** Coefficients and 95% credible intervals from Bayesian linear mixed models fitted to log FFD, GD, and TVT calculated from data downsampled to 125, 50, and 31.25 Hz using the average algorithm, with only one block per participant included

	FFD		GD		TVT	
Variable	b	CI	b	CI	b	CI
*31.25 Hz*						
(Intercept)	**5.28**	**5.23, 5.33**	**5.50**	**5.43, 5.57**	**5.81**	**5.70, 5.93**
Frequency	**0.038**	**0.001, 0.075**	**0.115**	**0.064, 0.166**	**0.181**	**0.123, 0.239**
*50 Hz*						
(Intercept)	**5.31**	**5.26, 5.35**	**5.58**	**5.51, 5.65**	**5.93**	**5.80, 6.05**
Frequency	0.014	-0.022, 0.048	**0.098**	**0.056, 0.141**	**0.161**	**0.104, 0.218**
*125 Hz*						
(Intercept)	**5.38**	**5.34, 5.42**	**5.68**	**5.61, 5.74**	**6.03**	**5.92, 6.15**
Frequency	0.018	-0.008, 0.043	**0.090**	**0.048, 0.131**	**0.159**	**0.105, 0.214**

Abbreviation: CI = 95% credible interval

Coefficients for which the 95% credible interval does not include 0 are shown in bold

**Table 12:** Number of subjects and experimental trials, total number of fixations (fix, detected by the Engbert & Kliegl algorithm), fixations per trial, and excluded/merged fixations (see Table 3 for details) in the data downsampled to 125, 50, and 31.25 Hz using the average and drop algorithms (all data included)

	Simulated sampling rate in Hz					
	Average algorithm			Drop algorithm		
	31.25	50	125	31.25	50	125
Number of subjects	32	32	32	32	32	32
Number of trials	12,800	12,800	12,800	12,800	12,800	12,800
Number of fix	152,359	174,213	185,760	160,369	173,525	183,943
Mean fix per trial	12.21	13.64	14.54	12.62	13.58	14.39
Fix < 80 ms	9711	3208	2926	7909	2454	2409
Fix < 80 ms (merged)	21	34	210	10	17	122
Fix > 800 ms	714	316	267	565	394	315
Fix before or after blink	268	2182	6686	944	3213	7261
Total fix excluded	10,663	5587	9635	9343	5919	9748
Percent of fix excluded	7	3.21	5.19	5.83	3.41	5.3

**Table 13:** Data downsampled using the drop algorithm, all blocks included: Mean and standard error for first fixation duration (FFD), gaze duration (GD), and total viewing time (TVT) on the target word by frequency condition and simulated sampling rate

	*FFD*		*GD*		*TVT*	
	Mean	SE	Mean	SE	Mean	SE
*31.25 Hz*						
High frequency	212	1.24	271	1.91	386	3.48
Low frequency	224	1.36	307	2.31	446	3.90
Effect	12		36		60	
*50 Hz*						
High frequency	222	1.11	294	1.93	425	3.66
Low frequency	231	1.20	328	2.36	485	4.08
Effect	9		34		60	
*125 Hz*						
High frequency	232	1.05	316	2.01	457	3.84
Low frequency	239	1.10	352	2.45	521	4.24
Effect	7		36		64	

All measures in ms

#### Downsampled data

##### One block per subject

*Drop algorithm*

As described in the Method section, we first downsampled the data using the “drop” algorithm, i.e., we “dropped” most of the samples from the data, keeping only every n-th sample in the data in order to simulate lower sampling rates of 125, 50, and 31.25 Hz. We did this for the entire data set (i.e., all four blocks), however, in order to keep the number of observations comparable to the analyses presented above, we randomly selected one block for each participant to include in our initial analysis. Table 7 shows the total number of subjects, trials, fixations, and the number of excluded fixations for each simulated sampling rate and the two downsampling algorithms (see below for details on the “average” algorithm).

We see that the simulated sampling rate affects fixation detection to some degree: there are fewer fixations overall detected at 31.25 Hz, and more fixations are excluded for being too short and too long. On the other hand, more fixations are detected at 125 Hz, but also many more are excluded for being within 100 ms of a blink. This may be because, at very low sampling rates, there are not enough samples left to detect fixations that were truncated by blinks. At 125 Hz, the number of fixations removed due to blinks is roughly in line with that at the non-simulated, higher sampling rates (see Table 3). Overall, therefore, we find that reducing the sampling rate clearly has an effect on fixation detection, with changes in how many short and long fixations are detected and whether fixations are detected as being close to blinks, but, even at these lower sampling rates, we observed that the number of fixations overall and fixations per trial is quite similar to that obtained at the original sampling rates. All of this indicates that the downsampled data are still a reasonable, if slightly degraded, representation of the eye-movement record.

As we did for the original data, we calculated FFD, GD, and TVT on the target word for the 400 sentences included in the analysis. In addition to the individual fixation exclusions shown in Table 7, we also again excluded very high gaze durations (GD > 1500 ms) and total viewing times (TVT > 2000 ms). The maximum number of observations excluded was in TVT at 125 Hz, with 41 of 3037 observations excluded (1.35%). Table 8 shows the mean and standard error for FFD, GD, and TVT on the target word by frequency condition and simulated sampling rate, with only one block included per subject, while Fig. 2 shows the distributions of FFD, GD, and TVT on the target word in the high and low-frequency conditions in the same data. We can see that at very low sampling rates, the distributions of fixation durations become more discrete. This is especially obvious in FFD at 31.25 Hz, as here the smallest difference in fixation duration that can be detected is 32 ms. The corresponding increase in noise in the eye-movement record is reflected to some degree in the standard errors of the means: for FFD, they were generally larger than in the original data. As expected, this increase in the standard error was greatest at 31.25 Hz and lowest at 125 Hz. However, we do not see the same pattern with the aggregated measures of GD and TVT. It appears that measures that combine more than one fixation duration are, to some degree, more robust to an increase in noise in individual fixation duration. The general shape of the distributions stays the same across the different sampling rates, and we can still observe the frequency effect in all sampling rates.

We performed the same analyses for the downsampled data with one block per subject as the ones reported above (Table 9). Here, the consequences of the increase in variability in the FFD measure is immediately obvious, as there is not enough evidence to detect the frequency effect at 125 Hz (*b* = 0.016, SE = 0.014, 95% CI [-0.012, 0.044]), 50 Hz (*b* = 0.029, SE = 0.015, 95% CI [-0.001, 0.058]), or 31.25 Hz (*b* = 0.031, SE = 0.017, 95% CI [-0.003, 0.065]), with all 95% CIs including 0. In contrast, the effect of the frequency manipulation on GD is clear at all sampling rates: 125 Hz (*b* = 0.09, SE = 0.02, 95% CI [0.05, 0.13]), 50 Hz (*b* = 0.1, SE = 0.02, 95% CI [0.06, 0.14]), and 31.25 Hz (*b* = 0.1, SE = 0.02, 95% CI [0.05, 0.15]). The same is true for the frequency effect on TVT at 125 Hz (*b* = 0.15, SE = 0.03, 95% CI [0.1, 0.21]), 50 Hz (*b* = 0.17, SE = 0.03, 95% CI [0.11, 0.22]), and 31.25 Hz (*b* = 0.17, SE = 0.03, 95% CI [0.11, 0.22]). This is due to both the much greater size of the frequency effect in GD and TVT and the robustness of aggregated measures to variability in the individual fixation durations we mentioned above.

*Average algorithm*

As described above, the drop algorithm may not be a perfect representation of how a lower-quality image sensor records data. Rather than collecting data during a short, precise interval, such a sensor is likely to accumulate data for a longer period of time, resulting in the data being “blurred” across the sampling period. A more accurate representation of this process may be to reduce the number of samples by averaging all the samples during a particular period rather than dropping all samples except for one.

As Table 7 shows, the number of detected and excluded fixations was quite similar for the “average” algorithm compared to the “drop” algorithm. There seemed to be a tendency for more short fixations to be detected in the “average” algorithm. Just as for the “drop” algorithm data, we calculated fixation time measures and excluded very high gaze durations (GD > 1500 ms) and total viewing times (TVT > 2000 ms). The maximum number of observations excluded was in TVT at 125 Hz, with 43 of 3038 observations excluded (1.42%).

Table 10 shows means and standard errors for the fixation time measures calculated from data downsampled by this “average” algorithm, and Fig. 3 shows the distributions of FFD, GD, and TVT on the target word in the high- and low-frequency conditions in the same data. The results are very similar to the means and distributions obtained from the “drop” algorithm data.

The results from the Bayesian linear mixed models on the data that were downsampled using the average algorithm (Table 11) were quite similar to those using the data downsampled using the drop algorithm, with one exception: there was not evidence for the frequency effect on FFD at 125 Hz (*b* = 0.018, SE = 0.013, 95% CI [-0.008, 0.043]) or 50 Hz (*b* = 0.014, SE = 0.018, 95% CI [-0.022, 0.048]), however, surprisingly, the 95 % CI at 31.25 Hz narrowly excluded 0 (*b* = 0.038, SE = 0.019, 95% CI [0.001, 0.075]). In some cases, averaging over samples can reduce the variability in each individual sample. In any case, we can conclude that at 3200 observations (1600 per condition), the frequency effect in FFD could not be detected consistently. On the other hand, we again found an effect of the frequency manipulations at all sampling rates in GD: 125 Hz (*b* = 0.09, SE = 0.02, 95% CI [0.05, 0.13]), 50 Hz (*b* = 0.1, SE = 0.02, 95% CI [0.06, 0.14]), and 31.25 Hz (*b* = 0.11, SE = 0.03, 95% CI [0.06, 0.17]). The same was true for TVT at 125 Hz (*b* = 0.16, SE = 0.03, 95% CI [0.1, 0.21]), 50 Hz (*b* = 0.16, SE = 0.03, 95% CI [0.1, 0.22]), and 31.25 Hz (*b* = 0.18, SE = 0.03, 95% CI [0.12, 0.24]). The fact that these results are nearly identical to those from the drop algorithm suggests that, despite the slight differences in terms of excluded fixations, the algorithm used to downsample the data does not matter much when it comes to detecting the effect of word frequency. In summary, we were able to detect the frequency effect on GD and TVT consistently even at very low sampling rates and with 1600 observations per condition. However, this was not true for FFD, which suggests that a larger sample size may be necessary to detect smaller effects and effects in FFD consistently.

##### All blocks included

In our second analysis, we downsampled all blocks to the same sampling rate and included them in the analysis. By doing this, we were able to use all the data collected and effectively have a much larger sample size (400 observations per participant, translating to 12,800 observations in total and 6400 per condition). Table 12 shows the total number of subjects, trials, fixations, and the number of excluded fixations for each simulated sampling rate when all blocks were downsampled and included in the analysis. As expected, the overall statistics for the full data set are quite similar to the data sets, including only one block per subject: depending on the algorithm and sampling rate used, between between 3 and 7% of fixations were excluded or merged for being too short or too long. There were no major differences between the two algorithms, but, just as in the data set with only one block per subject, there tended to be more fixations detected at higher simulated sampling rates.

*Drop algorithm*

We calculated FFD, GD, and TVT on the target word for all observations in all blocks in the data downsampled by the drop algorithm. In addition to the individual fixation exclusions shown in Table 12, we also again excluded very high gaze durations (GD > 1500 ms) and total viewing times (TVT > 2000 ms). The maximum number of observations excluded was in TVT at 125 Hz, with 152 of 12040 observations excluded (1.26%). The SEs make the impact of the larger sample size immediately obvious: they are much smaller than in the data sets with only one block per subject. The effect size estimates are generally similar to those in the analyses with one block per subject, with more consistency in GD and TVT and slightly more variability in FFD.

**Fig. 4:**
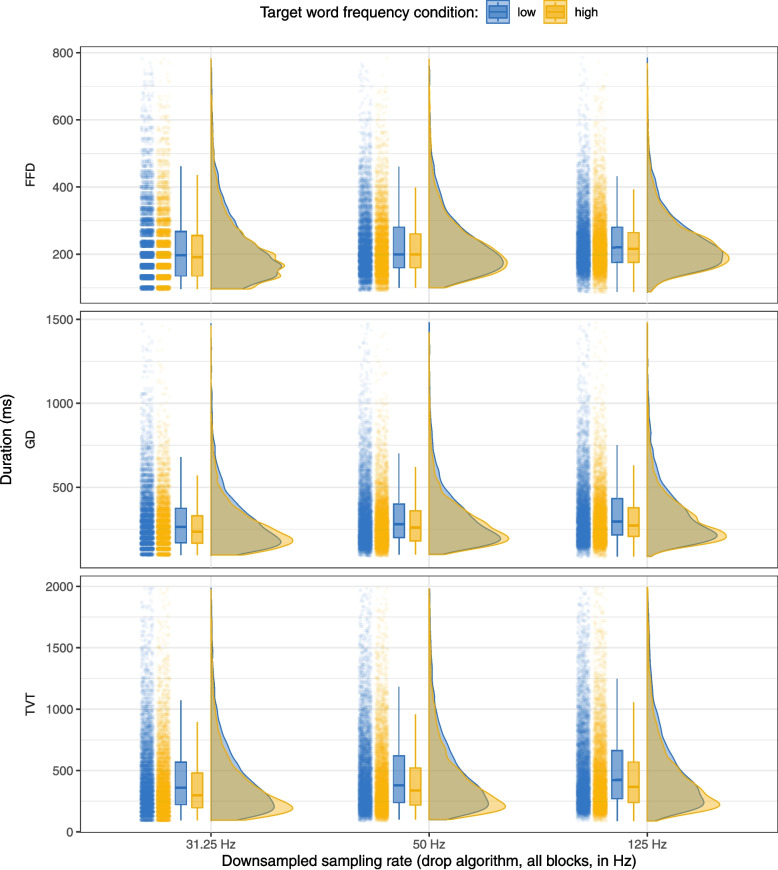
Data downsampled using the drop algorithm, all blocks included: Raincloud plot showing the distributions of first fixation duration (FFD), gaze duration (GD), and total viewing time (TVT) on the target word by frequency condition and simulated sampling rate. For each sampling rate, the left side of the plot shows a jittered point cloud and boxplots (representing median and IQR), while the right side shows half-violin plots of the density distributions

Table 13 shows the mean and standard error for first fixation duration (FFD), gaze duration (GD), and total viewing time (TVT) for the two frequency conditions calculated from the downsampled data for all blocks at the three simulated sampling rates, and Fig. 4 shows the distributions of FFD, GD, and TVT on the target word in the high- and low-frequency conditions in the same data. We performed the same analyses for the downsampled data as the ones reported above.

In the results from the Bayesian LMMs, the impact of the higher sample size is again immediately obvious (Table 14): in the data downsampled with the drop algorithm, we found evidence for the frequency effect on FFD at 125 Hz (*b* = 0.02, SE = 0.01, 95% CI [0.01, 0.04]), 50 Hz (*b* = 0.03, SE = 0.01, 95% CI [0.02, 0.05]), and at 31.25 Hz (*b* = 0.05, SE = 0.01, 95% CI [0.03, 0.06]). There was again, as expected, a clear effect of the frequency manipulation on gaze duration in the downsampled data at all sampling rates: 125 Hz (*b* = 0.09, SE = 0.01, 95% CI [0.07, 0.11]), 50 Hz (*b* = 0.09, SE = 0.01, 95% CI [0.07, 0.11]), and 31.25 Hz (*b* = 0.11, SE = 0.01, 95% CI [0.08, 0.13]). The same was true for TVT at 125 Hz (*b* = 0.14, SE = 0.02, 95% CI [0.11, 0.18]), 50 Hz (*b* = 0.15, SE = 0.02, 95% CI [0.11, 0.18]), and 31.25 Hz (*b* = 0.15, SE = 0.02, 95% CI [0.11, 0.18]). This suggests that, given a sufficiently large sample, we can even detect smaller and more subtle effects at low sampling rates.

*Average algorithm*

Just as for the analysis with only one block, the analysis with all blocks included showed very similar results for the “average” algorithm compared to the “drop” algorithm.

**Fig. 5:**
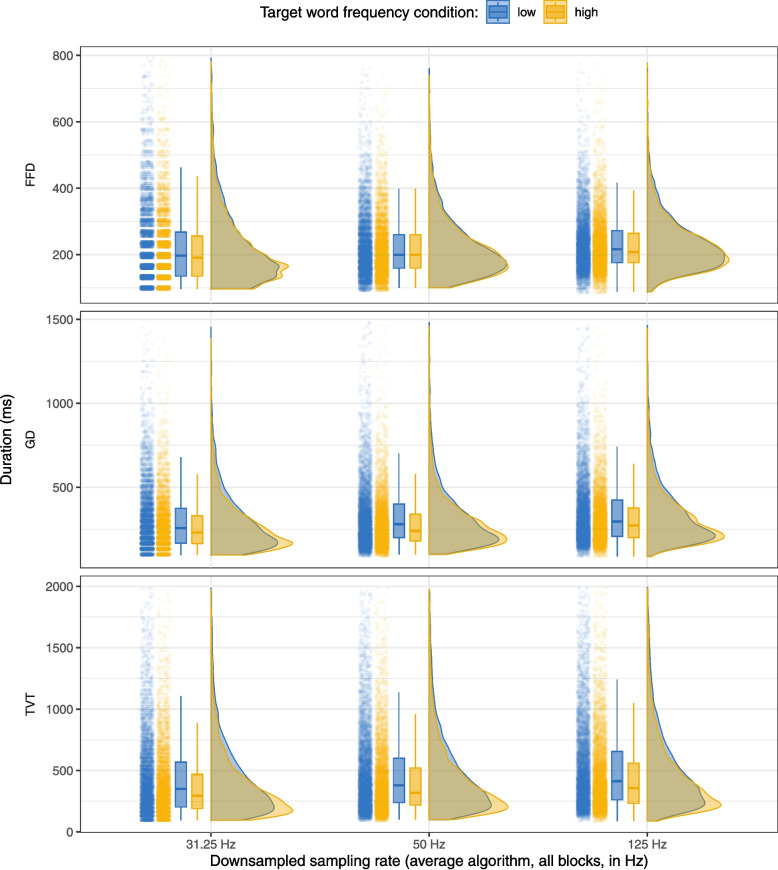
Data downsampled using the average algorithm, all blocks included: Raincloud plot showing the distributions of first fixation duration (FFD), gaze duration (GD), and total viewing time (TVT) on the target word by frequency condition and simulated sampling rate. For each sampling rate, the left side of the plot shows a jittered point cloud and boxplots (representing median and IQR), while the right side shows half-violin plots of the density distributions

We calculated FFD, GD, and TVT on the target word for all observations in all blocks in the data downsampled by the average algorithm (Table 15 and Fig. 5). In addition to the individual fixation exclusions shown in Table 12, we also again excluded very high gaze durations (GD > 1500 ms) and total viewing times (TVT > 2000 ms). The maximum number of observations excluded was in TVT at 125 Hz, with 143 of 12054 observations excluded (1.19%).

The results from the Bayesian linear mixed models on the data that were downsampled using the average algorithm (Table 16) were again very similar to those using the data downsampled using the drop algorithm, with one exception: we found evidence for the frequency effect on FFD at 125 Hz (*b* = 0.03, SE = 0.01, 95% CI [0.01, 0.04]) and 50 Hz (*b* = 0.03, SE = 0.01, 95% CI [0.01, 0.04]), but not at 31.25 Hz (*b* = 0.02, SE = 0.02, 95% CI [-0.01, 0.06]). The frequency effect was again detected at all sampling rates in GD: 125 Hz (*b* = 0.09, SE = 0.01, 95% CI [0.07, 0.11]), 50 Hz (*b* = 0.09, SE = 0.01, 95% CI [0.07, 0.11]), and 31.25 Hz (*b* = 0.1, SE = 0.02, 95% CI [0.05, 0.15]). The same was true for TVT: 125 Hz (*b* = 0.14, SE = 0.02, 95% CI [0.11, 0.18]), 50 Hz (*b* = 0.15, SE = 0.02, 95% CI [0.11, 0.18]), and 31.25 Hz (*b* = 0.16, SE = 0.03, 95% CI [0.11, 0.21]). The finding that we could not detect the frequency effect on FFD at 31.25 Hz in the data downsampled using the average algorithm suggests that, at very low sampling rates, small effects on FFD may still not be consistently detectable even at considerably higher sample sizes. However, the frequency effect was consistently detected in FFD at 50 and 125 Hz. In summary, we found the frequency effect on GD and TVT consistently in all sampling rates, both actual sampling rates provided by the eye-tracker and simulated sampling rates as low as 31.25 Hz. On FFD, the same effect was consistently detected at higher sampling rates (1000 and 2000 Hz) with a sample size of 1600/condition and at 50 and 125 Hz with a larger sample size of 6400/condition.

**Table 14:** Coefficients and 95% credible intervals from Bayesian linear mixed models fitted to log FFD, GD, and TVT calculated from data downsampled to 125, 50, and 31.25 Hz using the drop algorithm, with all blocks included in the analysis

	FFD		GD		TVT	
Variable	b	CI	b	CI	b	CI
*31.25 Hz*						
(Intercept)	**5.30**	**5.24, 5.35**	**5.54**	**5.46, 5.61**	**5.83**	**5.71, 5.95**
Frequency	**0.046**	**0.029, 0.063**	**0.105**	**0.080, 0.130**	**0.149**	**0.114, 0.184**
*50 Hz*						
(Intercept)	**5.35**	**5.30, 5.40**	**5.62**	**5.55, 5.69**	**5.94**	**5.82, 6.05**
Frequency	**0.034**	**0.019, 0.049**	**0.088**	**0.067, 0.109**	**0.145**	**0.112, 0.178**
*125 Hz*						
(Intercept)	**5.41**	**5.37, 5.45**	**5.70**	**5.63, 5.77**	**6.02**	**5.90, 6.14**
Frequency	**0.025**	**0.011, 0.038**	**0.087**	**0.068, 0.106**	**0.142**	**0.109, 0.175**

Abbreviation: CI = 95% credible interval

Coefficients for which the 95% credible interval does not include 0 are shown in bold

**Table 15:** Data downsampled using the average algorithm, all blocks included: Mean and standard error for first fixation duration (FFD), gaze duration (GD), and total viewing time (TVT) on the target word by frequency condition and simulated sampling rate

	*FFD*		*GD*		*TVT*	
	Mean	SE	Mean	SE	Mean	SE
*31.25 Hz*						
High frequency	212	1.32	268	1.96	378	3.50
Low frequency	224	1.44	305	2.37	440	3.96
Effect	11		37		62	
*50 Hz*						
High frequency	214	1.06	286	1.91	413	3.58
Low frequency	222	1.15	321	2.37	474	4.04
Effect	8		35		61	
*125 Hz*						
High frequency	227	1.01	312	2.00	453	3.82
Low frequency	234	1.05	347	2.43	517	4.25
Effect	6		35		64	

All measures in ms

## Discussion

In the present study, we tested how the sampling rate used to record eye movements affected our ability to detect the word frequency effect, a benchmark effect demonstrating the effect of cognitive processing on eye movements during reading, and to estimate its magnitude. Overall, we were able to detect the word frequency effect at all the sampling rates in all of the fixation time measures we calculated (with a few exceptions which we will discuss below). This was even true for the very low sampling rates we simulated, and demonstrates that, given a sufficient sample size, a low sampling rate is not necessarily an obstacle to investigating certain cognitive processes during reading. Importantly, the lowest simulated sampling rates we investigated (31.25 and 60 Hz) are within the range of consumer webcams, raising the possibility that useful eye-tracking data might be collected even using such low-cost devices. The few instances where we failed to find enough evidence to detect the effect give us useful insights into which fixation time measures are more or less robust to lower sampling rates.

In general, the word frequency effects we observed were in line with the literature across all sampling rates, although, in FFD, the effect of the word frequency manipulation on the target word was, at 3–12 ms, smaller than that observed by Rayner and Duffy (1986) and slightly smaller than that observed by Inhoff and Rayner (1986). Importantly, the data sets collected at the lower sampling rates did not show larger standard deviations and standard errors in the fixation time measures (suggesting that lowering the sampling rate does not result in more noisy fixation time measures overall).

Our results coincide with the predictions and results from Andersson et al. (2010): as long as there are sufficient observations (participants and trials), the mean and standard deviation of fixation duration estimates are not affected by lower sampling rates. Andersson et al. (2010) found the same when reducing sampling rate from 1250 to 250 and 50 Hz (using an algorithm similar to our drop algorithm on a small sample of reading data from ten participants). As predicted by Andersson et al. (2010), we did see a slight increase in the standard errors in the downsampled data at very low sampling rates, though only for FFD. It appears that, since they are a result of aggregating multiple fixation durations, GD and TVT are much more robust to increased noise in the fixation detection algorithm due to sampling error.

**Table 16:** Coefficients and 95% credible intervals from Bayesian linear mixed models fitted to FFD, GD, and TVT calculated from data downsampled to 125, 50, and 31.25 Hz using the “average” algorithm, with all blocks included in the analysis

	FFD		GD		TVT	
Variable	b	CI	b	CI	b	CI
*31.25 Hz*						
(Intercept)	**5.28**	**5.23, 5.33**	**5.54**	**5.47, 5.61**	**5.83**	**5.72, 5.94**
Frequency	0.024	-0.011, 0.059	**0.098**	**0.049, 0.146**	**0.162**	**0.112, 0.212**
*50 Hz*						
(Intercept)	**5.32**	**5.27, 5.37**	**5.59**	**5.52, 5.66**	**5.91**	**5.79, 6.03**
Frequency	**0.028**	**0.013, 0.043**	**0.092**	**0.070, 0.114**	**0.145**	**0.111, 0.180**
*125 Hz*						
(Intercept)	**5.39**	**5.35, 5.43**	**5.69**	**5.62, 5.75**	**6.01**	**5.89, 6.13**
Frequency	**0.027**	**0.014, 0.040**	**0.086**	**0.066, 0.106**	**0.143**	**0.109, 0.177**

Abbreviation: CI = 95% credible interval

Coefficients for which the 95% credible interval does not include 0 are shown in bold

Additionally, both the standard errors and means were very similar between the drop and average algorithms, suggesting that, at least for the purpose of detecting the frequency effect, it does not matter much whether we assume that low-sampling rate devices take precise snapshots of the gaze position or average all the positions during the sampling interval.

We did find that the effect size estimates differed somewhat numerically between the data sets collected at different sampling rates, and, although our analysis across all sampling rates did not show conclusive evidence for sampling rate affecting the size of the frequency effect, we failed to find sufficient evidence for the word frequency effect on FFD at 250 Hz. The same was true for the frequency effect on FFD at the simulated lower sampling rates when only analyzing 100 trials per subject (1600 observations per condition). However, the results of our analyses with 400 trials per subject (6400 observations per condition) show that this limitation can largely be overcome by increasing the sample size: with 400 observations per participant, we were able to detect the effect consistently even on FFD (with the exception of 31.25 Hz when using the average algorithm).

This, together with our analysis across sampling rates where we did not find evidence of an interaction between sampling rate and the frequency effect, shows that the main issue at low sampling rates is a decrease in power rather than a systematic inability to detect small effects. Our observation that the 250, 500, and 1000 Hz data sets are quite similar in terms of the number of fixations detected and the number of exclusions (as seen in Table 3) is also consistent with this conclusion. There does seem to be a small, but systematic difference in saccade and fixation detection between the 2000-Hz data and the other data sets, with more short fixations being detected and subsequently excluded or merged. As mentioned above, this may be an issue with the saccade detection algorithm splitting longer fixations into several shorter ones (perhaps because it is more sensitive to microsaccades), something that does not seem to occur at the lower sampling rates^6^. An adjustment to the saccade detection algorithm for 2000-Hz data may be able to address this issue. Additionally, the SR Research specifications state that the spatial noise when recording at 2000 Hz is higher than at 1000 Hz, which may also affect the fixation detection algorithm. In any case, we had no issues detecting the frequency effect at 2000 Hz.

**Table 17:** Sample size recommendations for detecting small effects in eye-tracking data based on our results

	Measure and effect size		
Sampling rate	FFD (5–10 ms)	GD (30 ms)	TVT (30 ms)
*High-precision eye-trackers (e.g., EyeLink)*			
1000 Hz	2400	1600	1600
250 Hz	3200	2400	2400
125 Hz	6000	3200	3200
30 Hz	9000	3200	3200
*Low-precision eye-trackers (e.g., Pupil Labs Core)*			
250 Hz	NR^*1*^	4800	4800
125 Hz	NR^*1*^	6400	6400
30 Hz	NR^*1*^	6400	6400

^*1*^NR = Not recommended

Recommended sample sizes are given as number of observations per condition. The desired sample size can be reached by increasing the number of subjects, the number of items, or both

Based on our results, we can make some recommendations along the lines of the rule of thumb proposed by Brysbaert and Stevens (2018), who (as mentioned above) suggested that, in a lexical decision task, an effect of 15 ms can be reliably detected if there are at least 1600 observations per condition. For eye-tracking in reading, we can conclude that 1600 observations per condition are sufficient to detect an effect of 30 ms in GD and TVT at all sample sizes. In fact the effect of the frequency manipulation was detected so consistently in these measures that we could speculate that even lower sample sizes may be sufficient. This was not the case for FFD. We observed a much smaller frequency effect of about 5–10 ms in FFD. Finding evidence for such a small effect was much more challenging. Adding to the issue is our observation that, at very low sampling rates, FFD, but not GD and TVT, seems to become noisier. This is likely due to FFD being calculated from individual fixations rather than it being the sum of multiple fixations. We did not calculate single-fixation duration in the present study, but we would assume that it would show similar effects. On the other hand, other aggregated measures such as go-past time are likely to be more robust, like GD and TVT. At a sampling rate of 1000 Hz, such a small effect of 5–10 ms in FFD may be detected with 1600 observations, but, given the variability in the estimates we observed across the different sampling rates, we would recommend a higher sample size of at least 2400 observations per condition. At lower sampling rates, and especially at sampling rates in the range of those we simulated (125 Hz and below), we would recommend a sample size of 6400 observations per condition when attempting to find small effects in FFD. The exception of this is the 31.25 Hz sampling rate, where we were unable to detect the effect of the target word frequency manipulation in the data downsampled using the average algorithm. We would therefore recommend that researchers who use a sampling rate of under 50 Hz collect at least 9000 observations per condition (40% more than the 6400 we collected) if they attempt to find evidence of an effect of 10 ms or less in FFD. At a sampling rate of 50 Hz or more, 6000 observations per condition may be sufficient. Table 17 summarizes these recommendations. Note that these are basic rules of thumb in the spirit of Brysbaert and Stevens (2018), and a detailed power analysis, e.g., using the simr package (Green & MacLeod, 2016), may yield a more precise estimate of the sample size necessary, especially when looking for effects of different sizes. These sample sizes may seem very large and imply investing a significant amount of resources, but there are many situations in which the initial investment to buy a high-sampling rate eye-tracker is a more difficult obstacle to overcome than finding a large number of participants.

In this study, we investigated the impact of changes in temporal resolution, i.e., sampling rate, on the ability to detect and measure the effects of cognitive processing on eye-movements. As mentioned in the introduction, the other major potential source of variability is spatial accuracy and precision. The estimates above assume that we use an eye-tracker with limited sampling rate, but a spatial accuracy and precision similar to the EyeLink Portable Duo system we used to collect our data (average accuracy: $0.47^{\circ }$; average sample-to-sample RMS difference at 1000 Hz: $0.15^{\circ }$). In reality, more affordable eye-tracking systems are very likely to have lower accuracy and more spatial noise. For example, as we mentioned above, the Pupil Labs Core has a lower spatial accuracy and higher spatial noise than the EyeLink 1000 (Ehinger et al., 2019). In reading, errors in spatial accuracy may lead to fixations being counted as being on different words than the ones that were actually fixated, making it more difficult to detect word-related effects. Increased spatial noise may make fixation detection more difficult in general, leading to noisier estimates of fixation time. Because of this, we would suggest increasing the sample size at least by a factor of two when using a low spatial accuracy and precision eye-tracker. Table 17 reflects this adjustment. Future research with such devices will be needed to give more precise estimates. Note that, at this point, we do not recommend using low-precision eye-trackers to investigate effects in FFD and similar measures, such as single fixation duration, that are based on one fixation per word.

In conclusion, we have shown that low eye-tracker sampling rate, in itself, is not an obstacle to measuring the effects of cognitive processing on eye movements during reading and, in fact, only affects power slightly at very low sampling rates (125 Hz and below, particularly in FFD). Given that low-sampling rate devices are also likely to have lower spatial accuracy and precision, we recommend that researchers wishing to use such a device for reading research use large font sizes and large sample sizes in order to compensate for spatial noise. In principle, our results indicate that useful data on cognitive processes during reading can be obtained with a wide variety of eye-tracking devices. We hope that our results lead to a wider use of eye-tracking methods in the study of reading, especially in languages and countries that have been understudied so far (Angele & Duñabeitia, 2024). We recommend that researchers using eye-tracking devices with low sampling rates and low spatial accuracy and precision collect sufficiently large samples, employ simple designs (as few conditions as possible), and focus on aggregate fixation time measures such as GD and TVT.

## Data Availability

Materials, data files and R code for the analysis can be found at https://osf.io/hn5a2/.

## Footnotes

1 This is measured by taking the root mean square (RMS) of the angular distances between adjacent gaze position samples (sample-to-sample or S2S RMS) as an estimate of the spatial noise, although note that Ehinger et al. (2019) use 20%-winsorized means, effectively only taking into account the central 20% of the distribution.

2 $$\text {Zipf} \!=\! \log _{10}\left( \text {frequency\_per\_billion}\right) \!=\! \log _{10}\left( \text {frequency\_per\_million}\right) +3$$

3 The versions of R and all packages used are as follows: R (Version 4.4.3; R Core Team, 2024) and the R-packages *brms* (Version 2.22.0; Bürkner, 2017, 2018, 2021), *broom.mixed* (Version 0.2.9.6; Bolker & Robinson, 2024), *devtools* (Version 2.4.5; Wickham et al., 2022), *dplyr* (Version 1.1.4; Wickham et al., 2023), *forcats* (Version 1.0.0; Wickham, 2023a), *ggplot2* (Version 3.5.1; Wickham, 2016), *gt* (Version 0.11.1; Iannone et al., 2024; Sjoberg et al., 2021), *gtsummary* (Version 2.1.0; Sjoberg et al., 2021), *huxtable* (Version 5.6.0; Hugh-Jones, 2024), *kableExtra* (Version 1.4.0; Zhu, 2024), *lme4* (Version 1.1.36; Bates et al., 2015), *lubridate* (Version 1.9.4; Grolemund & Wickham, 2011), *Matrix* (Version 1.7.2; Bates et al., 2024), *openai* (Version 0.4.1; Rudnytskyi, 2023), *papaja* (Version 0.1.3; Aust & Barth, 2023), *purrr* (Version 1.0.4; Wickham & Henry, 2023), *qs* (Version 0.27.3; Ching, 2024), *Rcpp* (Eddelbuettel & Balamuta, 2018; Version 1.0.14; Eddelbuettel & François, 2011), *readr* (Version 2.1.5; Wickham et al., 2024), *rstan* (Version 2.32.7; Stan Development Team, 2024), *saccades* (Version 0.2.1; von der Malsburg, 2019), *simr* (Version 1.0.7; Green & MacLeod, 2016), *StanHeaders* (Version 2.32.10; Stan Development Team, 2020), *stringr* (Version 1.5.1; Wickham, 2023b), *tibble* (Version 3.2.1; Müller & Wickham, 2023), *tidyr* (Version 1.3.1; Wickham et al., 2024), *tidyverse* (Version 2.0.0; Wickham et al., 2019), *tinylabels* (Version 0.2.4; Barth, 2023), *usethis* (Version 3.1.0; Wickham et al., 2024) and *zoom* (Version 2.0.6; Barbu, 2014).

4 Please note that, throughout this article, the abbreviation CI will refer to Bayesian credible intervals and not frequentist confidence intervals.

5 The exact contrast specifications were as follows: Contrast 1: c(-.25, -.25, .25, .25); Contrast 2: c(-.5, .5, 0, 0); Contrast 3: c(0, 0, -.5, .5). The levels in the sampling rate factor were ordered as follows: 250, 500, 1000, and 2000 Hz

6 This also fits in with the preference of many reading researchers to collect eye-movement data at 1000 Hz rather than 2000 Hz.

## Code availability

Experiment code, R code for the analysis and the RMarkdown code for the manuscript can be found at https://osf.io/hn5a2/.

## Declarations

### Open Practices Statement

This experiment was pre-registered; the preregistration form can be found at https://osf.io/rqpke/, and the materials, data files and R code for the analysis can be found at https://osf.io/hn5a2/. The analyses of the down-sampled data were not pre-registered. The pre-registration above contains further analyses that we have not included in this manuscript due to space constraints. We hope to report these analyses in a future publication.
